# Hox genes mediate the escalation of sexually antagonistic traits in water striders

**DOI:** 10.1098/rsbl.2018.0720

**Published:** 2019-02-06

**Authors:** Antonin Jean Johan Crumière, Abderrahman Khila

**Affiliations:** Institut de Génomique Fonctionnelle de Lyon, Université de Lyon, Université Claude Bernard Lyon 1, CNRS UMR 5242, Ecole Normale Supérieure de Lyon, 46, allée d'Italie, 69364 Lyon Cedex 07, France

**Keywords:** Hox genes, sexual conflict, coevolution of the sexes, sexually antagonistic traits, development

## Abstract

Sexual conflict occurs when traits favoured in one sex impose fitness costs on the other sex. In the case of sexual conflict over mating rate, the sexes often undergo antagonistic coevolution and escalation of traits that enhance females' resistance to superfluous mating and traits that increase males' persistence. How this escalation in sexually antagonistic traits is established during ontogeny remains unclear. In the water strider *Rhagovelia antilleana,* male persistence traits consist of sex combs on the forelegs and multiple rows of spines and a thick femur in the rear legs. Female resistance traits consist of a prominent spike-like projection of the pronotum. RNAi knockdown against the Hox gene *Sex Combs Reduced* resulted in the reduction in both the sex comb in males and the pronotum projection in females. RNAi against the Hox gene *Ultrabithorax* resulted in the complete loss or reduction of all persistence traits in male rear legs. These results demonstrate that Hox genes can be involved in intra- and inter-locus sexual conflict and mediate escalation of sexually antagonistic traits.

## 1. Introduction

The evolutionary interests of males and females during mating interactions often diverge, leading to the coevolution of sexually antagonistic traits that are favoured in one sex at a fitness cost to the other [1,2]. Empirical and theoretical data on the coevolution of the sexes established sexual conflict as a major force in evolutionary change within and between lineages [1,3]. The consequences of sexually antagonistic selection are manifest in some spectacular shape changes in water striders, one of the most prominent model systems for the study of sexual antagonism in nature [4–7]. In many species, males are often favoured to mate repeatedly, but females pay increasing fitness costs for multiple mating [1,8]. The repeated evolution of grasping traits that allow males to overcome females' resistance is often matched by the evolution of anti-grasping traits in females that enhance their ability to resist [1,6,9,10]. Sexually antagonistic traits are highly variable in shape and can occur in any segment along the body axis. Examples involve the modification of antennae, forelegs or rear legs into grasping traits in the males, whereas females are known to match these with various anti-grasping traits such as erect abdominal spines [4,6,11].

Males and females share the same genome, and selection on sex-specific traits inevitably results in inter-locus or intra-locus sexual conflict [1,10,12,13]. Inter-locus sexual conflict occurs when males and females undergo different selective pressures at different loci, potentially leading to antagonistic coevolution [12,14]. On the other hand, intra-locus sexual conflict occurs when males and females undergo different selective pressures at the same locus, a process that is thought to limit the evolution of one sex owing to the change in the other [12,14]. Several studies have highlighted the role of developmental genes in shaping certain male-specific morphologies in insects, including a case of an antagonistic trait in water striders [6,15,16]. However, the developmental genetic dynamics underlying the escalation of sexually antagonistic traits in both sexes remain untested.

In the water strider *Rhagovelia antilleana*, both males and females have evolved antagonistic traits, which influence mating rate, along the body axis [5,17]. On the first thoracic segment, males develop sex combs located on their forelegs and winged females develop a prominent spike-like extension of the pronotum [5]. On the third thoracic segment, males have modified rear legs with rows of spines and large femurs, whereas females possess fewer spines and thinner femurs [5]. Because Hox genes are known to specify segment identity, we sought to test a possible role of these genes in the development of sexually antagonistic traits and whether these genes are involved in intra- and inter-locus sexual conflict. By inactivating *Sex Combs Reduced* (*Scr*) and *Ultrabithorax* (*Ubx*), known to control the identity of the first and third thoracic segments, respectively, we uncovered the importance of these two genes in sexual conflict and their role in the escalation of male and female sexually antagonistic traits.

## 2. Material and methods

### (a) Insect rearing

Laboratory populations of *R. antilleana* were kept in water tanks at 25°C, 50% humidity, 14 h of daylight and fed daily on crickets. Styrofoam floaters were provided for adult females to lay eggs on. Eggs were regularly transferred to separate tanks to prevent cannibalism on the newly hatched nymphs.

### (b) Cloning of *R. antilleana* *Scr* and *Ubx*

Extraction of total RNA from a mix of 10 complete *R. antilleana* first to fifth instar nymphs was performed using Trizol. cDNA was synthesized using SuperScript III First-Strand kit according to the manufacturer's instructions (Invitrogen). Fragments of *Scr* and *Ubx* genes were amplified (electronic supplementary material, figures S1 and S2) by PCR using GoTaq G2 DNA polymerase (Promega) and the primers described in electronic supplementary material, table S1. PCR products were examined by electrophoresis, purified using PCR Minelute kit (Qiagen) and cloned into a pGEM-T vector kit (Promega). The sequences of *R. antilleana Scr* and *Ubx* can be retrieved in GenBank using the following accession numbers: MG999826 and MG999808, respectively, and in electronic supplementary material, figures S1 and S2.

### (c) Nymphal RNA interference in *R. antilleana*

RNAi procedures have been efficiently adapted in water striders [6,18,19]. To synthesize double-stranded RNA, a DNA template was produced using PCR and *Scr* or *Ubx* primers tagged with a T7 RNA polymerase promoter and the *Scr* or *Ubx* plasmids as PCR templates (electronic supplementary material, figures S1 and S2, and table S1). The resulting PCR products were purified using Minelute kit (Qiagen) followed by an *in vitro* transcription with T7 RNA polymerase + (Ambion) to obtain the double-stranded RNA (dsRNA) corresponding to each gene. Both dsRNAs were purified using RNeasy Mini Kit (Qiagen), concentrated with speedvac and re-suspended in 1× injection buffer [20] at a final concentration of 3 µg µl^−1^. Yellow fluorescent protein (*yfp*) dsRNA was used as a control at 1.8 µg µl^−1^ concentration. We performed injections in *R. antilleana* first to third nymphal instars using a SteREO Discovery V8 (Zeiss), a Cell Tram Vario Oil Eppendorf injector and a Narishige micromanipulator under CO_2_ anaesthesia. The number of injected nymphs, emerged adults and frequency of successful knockdown are presented in electronic supplementary material, table S2.

### (d) Imaging and analysis of phenotypes

Image acquisition and observation of secondary sexual traits were performed using a SteREO Discovery V12 (Zeiss) and a ZEISS Merlin Compact Scanning Electron Microscope. Image analysis and measurements of the different body parts were performed using Zen software (Zeiss) and Fiji software [21]. For measurements of the teeth structures composing the sex combs, we measured the length of 20 teeth in three different males for both *yfp* control and *Scr* RNAi, which corresponds to an effective *yfp* control *n* = 60, *Scr* RNAi *n* = 60. Measurements of pronotum between control and *Scr* RNAi have been performed on: control *n* = 12 (4 *yfp* + 8 wild-type), *Scr* RNAi *n* = 7. Then measurements and quantification of *Ubx* RNAi phenotypes have been performed with: *yfp* control males *n* = 21, *Ubx* RNAi males *n* = 9, *yfp* control females *n* = 12, *Ubx* RNAi females *n* = 10.

### (e) Statistical analysis

Shapiro tests (to test for the normal distribution of data), Student's *t*-tests and Wilcoxon tests (depending whether variables followed normal distributions) were performed using RStudio version 1.0.153 for the statistical analysis of phenotypic quantification. R script used in this study [22] is available in the Dryad Digital Repository using the following link: http://dx.doi.org/10.5061/dryad.s76h0s6.

## 3. Results

### (a) *Scr* is required for both male persistence and female resistance traits

**Figure 1.**
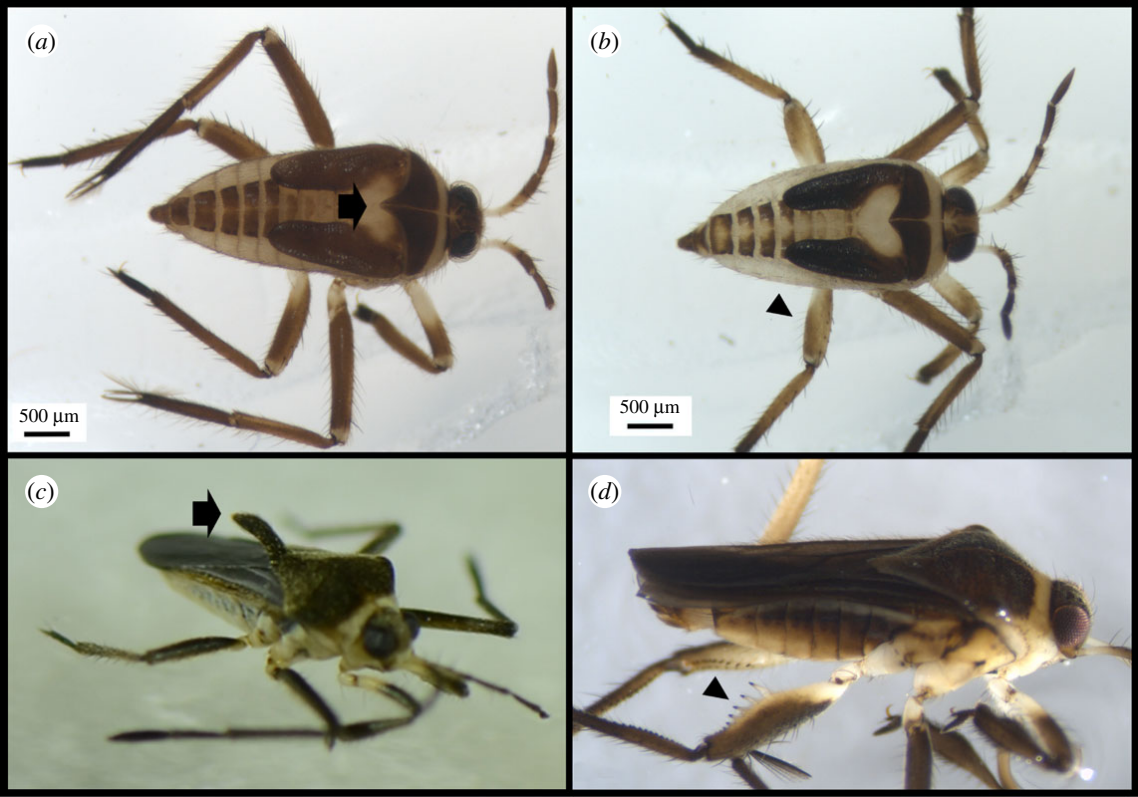
Development of male and female sexually antagonistic traits. (*a*) Fifth instar female nymph showing a slight increase in pronotum extension (arrow) compared to male fifth instar nymph in (*b*). (*b*) Male fifth instar nymph where modifications on the rear legs have not yet developed (arrowhead). (*c*) Adult winged female showing the pronotum projection (arrow), which is absent in adult males in (*d*). Males (*d*), however, have large spines and thicker rear leg femurs (arrowhead).

**Figure 2.**
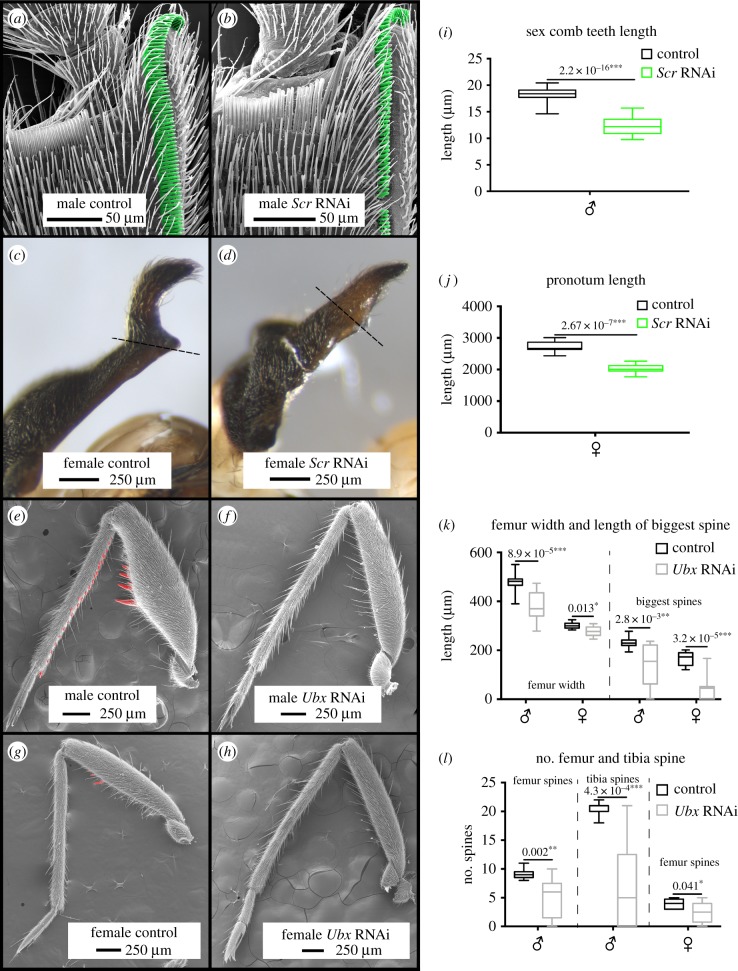
*Scr* and *Ubx* RNAi knockdown phenotypes. In *R. antilleana* males, *Scr* RNAi induces a reduction in the size of the teeth that compose the sex comb (*a,b,i*). In *R. antilleana* females, *Scr* RNAi induces the reduction in the size and a modification of the shape of the pronotum projection (*c,d,j*). In *R. antilleana* males, *Ubx* RNAi induces a reduction in the size of the rear leg femur and a reduction or loss of the spikes present on the femur and on the tibia (*e,f,k*). In females, *Ubx* RNAi induces similar modifications (*g,h,l*). *p*-values are indicated on top of each comparison. Sample sizes are indicated in §2. *p*-values: * < 0.05, ** < 0.01, *** < 0.001.

Secondary sexual traits in *R. antilleana* males and females start to appear late during the fifth nymphal instar (figure 1*a,b*), and only become prominent in the adult (figure 1*c,d*). In the adults, male forelegs are equipped with sex combs (figure 2*a*) [5], whereas female pronotum exhibits a prominent spike-like projection (figures 1*c* and 2*c*) [5]. The sex combs and the pronotum projection are both located on the first thoracic segment. Because this segment is under the control of the Hox gene *Scr* [16,23,24], we tested the role of *Scr* in the development of these structures. In males, *Scr* RNAi caused a notable reduction in the size of the teeth forming the comb (figure 2*b,i*). Interestingly, in females, *Scr* RNAi also resulted in a reduction in the size and disruption of the shape of the pronotum projection (figure 2*d,j*). These results demonstrate that the same Hox gene, *Scr*, is involved in the development of both male persistence and female resistance traits that are located in its domain of action, i.e. the first thoracic segment.

### (b) *Ubx* shapes another set of male persistence traits found in the rear legs

The rear legs of *R. antilleana* males are equipped with sets of large and small spines arranged in rows on the trochanter, femur and tibia (figure 2*e*) [5]. We therefore tested the role of the Hox gene *Ultrabithorax,* which is known to specify the identity of the third thoracic segment in insects [25–30]. Nymphal RNAi knockdown against *Ubx* resulted in a specific loss or reduction in all the armaments that otherwise develop on male rear legs (figure 2*e*,*f*) without any effect on the other segments of the legs (electronic supplementary material, figure S3). Specifically, the width of the femur was significantly reduced and the spines on both femur and tibia were lost or reduced such that the rear leg of the male now resembled that of the female (figure 2*e,f,k,l*). In females, *Ubx* RNAi also induced the loss of the small spines in the femur (figure 2*g,h,k,l*), suggesting that correlation of *Ubx* expression is associated with the slight modifications of female rear legs. These results indicate that *Ubx* mediates the development of male persistence traits located in the third thoracic segment in *R. antilleana*.

## 4. Discussion

We have reported that antagonistic coevolution of the sexes, here in *Rhagovelia antilleana*, can be developmentally controlled by the sex-specific action of the highly pleiotropic Hox genes. Our results also uncover, for the first time, a case where the same gene, here *Scr*, controls the development of male persistence (sex comb) and female resistance (pronotum projection) traits [5]. This indicates an ongoing intra-locus conflict at the *Scr* locus and suggests that sexual conflict over mating rate can be associated with intra-locus sexual conflict [1,14]. How this opposing role of *Scr* is mediated between the sexes of *R. antilleana* is unknown. Although both male and female traits are located in the first thoracic segments, the sex combs being in the leg and the projection being at the posterior portion of the pronotum indicate that these traits develop from distinct cell populations. It is therefore likely that alleles controlling the sex-specific regulation of *Scr* mediate its dimorphic expression and function, leading to sex-specific antagonistic traits.

*Ubx*, on the other hand, controls the elaboration of male rear legs, which increases male persistence [5]. This suggests an ongoing inter-locus conflict between *Ubx* and *Scr* owing to sexually antagonistic coevolution between males and females of this species. Inter-locus conflict is expected to generate dimorphism and fuel arms races because of different loci under divergent selective pressures, while intra-locus conflict is expected to slow the evolution of the sexes because of the shared loci under divergent selective pressures [12,14]. Our results provide a new perspective on antagonistic coevolution because both inter- and intra-locus conflicts at the *Scr* and *Ubx* loci participate in sexual dimorphism.

How these evolutionarily constrained genes can shape sex-specific morphologies during development remains unknown. Hox genes are known to control a large number of downstream targets that can diverge greatly among insects [25,31]. It is likely that *Ubx* and *Scr* mediate the development of sexually antagonistic traits by interacting with sex-specific targets, as is the case for some fly sex-specific phenotypes [32,33]. The late development of sexually antagonistic traits may have favoured changes in Hox targets to accumulate without any pleiotropic effects, thus favouring rapid changes in Hox function. Other mechanisms could explain the sex-specific function of Hox genes in *R. antilleana*. Interactions with sex-determination gene isoforms, such as *doublesex* (*dsx*) [16,34,35], would allow the generation of sex-specific structures by differentially regulating Hox genes at the cellular level [16,32], which in turn could also differentially regulate *dsx* in both sexes [16,35]. These interactions and regulations might allow Hox transcription factors to be expressed in different cell populations within the same segment [16,32] and are consistent with sex-specific gene regulation participating in intra-locus sexual conflict [12,36]. Testing these hypotheses should further improve our understanding of the developmental genetic basis of antagonistic coevolution and the underlying role of Hox genes and their sex-specific regulation.

## Supplementary Material

Figures S1 - S3;Tables S1 and S2

## Data accessibility

Sequences of *Rhagovelia antilleana Ultrabithorax* and *Sex* combs reduced can be retrieved in GenBank under the following accession numbers: *Ultrabithorax*: MG999808; *Sex combs reduced*: MG999826. Data and R script [22] used for the analysis performed in this study are available on the Dryad Digital Repository: http://dx.doi.org/10.5061/dryad.s76h0s6.

## References

[1] Arnqvist G, Rowe L 2005 Sexual conflict, xii, 330 p Princeton, NJ: Princeton University Press.

[2] Rice WR, Holland B 1997 The enemies within: intergenomic conflict, interlocus contest evolution (ICE), and the intraspecific Red Queen. Behav. Ecol. Sociobiol. 41, 1–10. (10.1007/s002650050357)

[3] Arnqvist G, Edvardsson M, Friberg U, Nilsson T 2000 Sexual conflict promotes speciation in insects. Proc. Natl Acad. Sci. USA 97, 10 460–10 464. (10.1073/pnas.97.19.10460) PMC27046 10984538

[4] Arnqvist G, Rowe L 2002 Correlated evolution of male and female morphologles in water striders. Evol. Int. J. Org. Evol. 56, 936–947. (10.1111/j.0014-3820.2002.tb01406.x) 12093029

[5] Crumière AJJ, Armisén D, Vargas-Lowman A, Kubarakos M, Moreira FFF, Khila A. 2018 Escalation and constraints of antagonistic armaments in water striders. bioRxiv (10.1101/430322)

[6] Khila A, Abouheif E, Rowe L 2012 Function, developmental genetics, and fitness consequences of a sexually antagonistic trait. Science 336, 585–589. (10.1126/science.1217258) 22556252

[7] Perry JC, Rowe L 2018 Sexual conflict in its ecological setting. Phil. Trans. R. Soc. B 373, 20170418 (10.1098/rstb.2017.0418) 30150217 PMC6125725

[8] Rowe L, Arnqvist G, Sih A, Krupa J 1994 Sexual conflict and the evolutionary ecology of mating patterns: water striders as a model system. Trends Ecol. Evol. 9, 289–293. (10.1016/0169-5347(94)90032-9) 21236857

[9] Parker GA 1983 Arms races in evolution—an ESS to the opponent-independent costs game. J. Theor. Biol. 101, 619–648. (10.1016/0022-5193(83)90019-X)

[10] Parker GA 1979 Sexual selection and sexual conflict. In Sexual selection and reproductive competition in insects (ed. Blum MSBNA), pp. 123–166. New York, NY: Academic Press.

[11] Westlake KP, Rowe L 1999 Developmental costs of male sexual traits in the water strider *Rheumatobates rileyi*. Revue canadienne de zoologie 77, 917–922. (10.1139/z99-058)

[12] Bonduriansky R, Chenoweth SF 2009 Intralocus sexual conflict. Trends Ecol. Evol. 24, 280–288. (10.1016/j.tree.2008.12.005) 19307043

[13] Chapman T, Arnqvist G, Bangham J, Rowe L 2003 Sexual conflict. Trends Ecol. Evol. 18, 41–47. (10.1016/S0169-5347(02)00004-6)

[14] Rowe L, Chenoweth SF, Agrawal AF 2018 The genomics of sexual conflict. Am. Nat. 192, 274–286. (10.1086/698198) 30016158

[15] Ledon-Rettig CC, Zattara EE, Moczek AP 2017 Asymmetric interactions between *doublesex* and tissue- and sex-specific target genes mediate sexual dimorphism in beetles. Nat. Commun. 8, 14593 (10.1038/ncomms14593) 28239147 PMC5333360

[16] Tanaka K, Barmina O, Sanders LE, Arbeitman MN, Kopp A 2011 Evolution of sex-specific traits through changes in HOX-dependent *doublesex* expression. PLoS Biol. 9, e1001131 (10.1371/journal.pbio.1001131) 21886483 PMC3160335

[17] Polhemus DA 1997 Systematics of the genus Rhagovelia Mayr (Heteroptera: Veliidae) in the Western Hemisphere (exclusive of the angustipes complex), ii, 386 p Lanham, MD: Entomological Society of America.

[18] Santos ME, Berger CS, Refki PN, Khila A 2015 Integrating evo-devo with ecology for a better understanding of phenotypic evolution. Brief Funct. Genomics 14, 384–395. (10.1093/bfgp/elv003) 25750411 PMC4652033

[19] Santos ME, Le Bouquin A, Crumière AJJ, Khila A. 2017 Taxon-restricted genes at the origin of a novel trait allowing access to a new environment. Science 358, 386–390. (10.1126/science.aan2748) 29051384

[20] Rubin GM, Spradling AC 1982 Genetic transformation of *Drosophila* with transposable element vectors. Science 218, 348–353. (10.1126/science.6289436) 6289436

[21] Schindelin J et al. 2012 Fiji: an open-source platform for biological-image analysis. Nat. Methods 9, 676–682. (10.1038/nmeth.2019) 22743772 PMC3855844

[22] Crumière AJJ, Khila A 2018 Data from: Hox genes mediate the escalation of sexually antagonistic traits in water striders Dryad Digital Repository. (10.5061/dryad.s76h0s6) PMC6405465 30958129

[23] Chesebro J, Hrycaj S, Mahfooz N, Popadic A 2009 Diverging functions of Scr between embryonic and post-embryonic development in a hemimetabolous insect, *Oncopeltus fasciatus*. Dev. Biol. 329, 142–151. (10.1016/j.ydbio.2009.01.032) 19382295 PMC2775506

[24] Kopp A, Duncan I, Godt D, Carroll SB 2000 Genetic control and evolution of sexually dimorphic characters in *Drosophila*. Nature 408, 553–559. (10.1038/35046017) 11117736

[25] Armisen D, Refki PN, Crumiere AJJ, Viala S, Toubiana W, Khila A 2015 Predator strike shapes antipredator phenotype through new genetic interactions in water striders. Nat. Commun. 6, 8153 (10.1038/ncomms9153) 26323602 PMC4568302

[26] Davis GK, Srinivasan DG, Wittkopp PJ, Stern DL 2007 The function and regulation of *Ultrabithorax* in the legs of *Drosophila melanogaster*. Dev. Biol. 308, 621–631. (10.1016/j.ydbio.2007.06.002) 17640629 PMC2040266

[27] Khila A, Abouheif E, Rowe L 2014 Comparative functional analyses of ultrabithorax reveal multiple steps and paths to diversification of legs in the adaptive radiation of semi-aquatic insects. Evolution 68, 2159–2170. (10.1111/evo.12444) 24766229

[28] Refki PN, Armisen D, Crumiere AJJ, Viala S, Khila A 2014 Emergence of tissue sensitivity to Hox protein levels underlies the evolution of an adaptive morphological trait. Dev. Biol. 392, 441–453. (10.1016/j.ydbio.2014.05.021) 24886828 PMC4111901

[29] Rozowski M, Akam M 2002 Hox gene control of segment-specific bristle patterns in *Drosophila*. Genes Dev. 16, 1150–1162. (10.1101/gad.219302) 12000797 PMC186253

[30] Stern DL 2003 The Hox gene *Ultrabithorax* modulates the shape and size of the third leg of *Drosophila* by influencing diverse mechanisms. Dev. Biol. 256, 355–366. (10.1016/S0012-1606(03)00035-6) 12679108

[31] Pavlopoulos A, Akam M 2011 Hox gene *Ultrabithorax* regulates distinct sets of target genes at successive stages of *Drosophila* haltere morphogenesis. Proc. Natl Acad. Sci. USA 108, 2855–2860. (10.1073/pnas.1015077108) 21282633 PMC3041078

[32] Barmina O, Kopp A 2007 Sex-specific expression of a HOX gene associated with rapid morphological evolution. Dev. Biol. 311, 277–286. (10.1016/j.ydbio.2007.07.030) 17868668

[33] Williams TM, Carroll SB 2009 Genetic and molecular insights into the development and evolution of sexual dimorphism. Nat. Rev. Genet. 10, 797–804. (10.1038/nrg2687) 19834484

[34] Rice G, Barmina O, Hu K, Kopp A 2018 Evolving *doublesex* expression correlates with the origin and diversification of male sexual ornaments in the *Drosophila immigrans* species group. Evol. Dev. 20, 78–88. (10.1111/ede.12249) 29372584 PMC6444933

[35] Wang W, Yoder JH 2012 Hox-mediated regulation of *doublesex* sculpts sex-specific abdomen morphology in *Drosophila*. Dev. Dyn. 241, 1076–1090. (10.1002/dvdy.23791) 22488883

[36] Mank JE, Wedell N, Hosken DJ 2013 Polyandry and sex-specific gene expression. Phil. Trans. R. Soc. B 368, 20120047 (10.1098/rstb.2012.0047) 23339238 PMC3576581

