# Supplementary material for: Hox genes mediate the escalation of sexually antagonistic traits in water striders
Source: Biol Lett. 2019 Feb 6;15(2):20180720. doi: 10.1098/rsbl.2018.0720 (PMC6405465; doi:10.1098/rsbl.2018.0720)
Supplement: Figures S1 - S3;Tables S1 and S2 [file rsbl20180720supp1.docx]

DNA:  atgagttcctaccagttcgttaactcgctggcttcctgctacggggggcagggcgggggc
+1fr: ·M··S··S··Y··Q··F··V··N··S··L··A··S··C··Y··G··G··Q··G··G··G·


DNA:  cgttctcccgtcgagccccaaagtcccgaatactacggtgccgcgcccggttacggctgt
+1fr: ·R··S··P··V··E··P··Q··S··P··E··Y··Y··G··A··A··P··G··Y··G··C·


DNA:  tattcgccgcagggatacgcgggctacgtgcaacagccgcctgtcgtggaatacgctcag
+1fr: ·Y··S··P··Q··G··Y··A··G··Y··V··Q··Q··P··P··V··V··E··Y··A··Q·


DNA:  ctcggttcgcaccaatcgttggctcaagcgggaccgaggatcggtcagctggctcccgta
+1fr: ·L··G··S··H··Q··S··L··A··Q··A··G··P··R··I··G··Q··L··A··P··V·


DNA:  cagcatcaacagcccagttgtaaattcgaagggctaggctctcctcaagatctgagcacg
+1fr: ·Q··H··Q··Q··P··S··C··K··F··E··G··L··G··S··P··Q··D··L··S··T·


DNA:  cagcccgcgagaagcgggagtccggcggcacccggattagccagcccacgggccggaccc
+1fr: ·Q··P··A··R··S··G··S··P··A··A··P··G··L··A··S··P··R··A··G··P·


DNA:  gcacctagccaagcgagcagctcacccgcttcaaccgcgtccacgtcaagccagcccgcc
+1fr: ·A··P··S··Q··A··S··S··S··P··A··S··T··A··S··T··S··S··Q··P··A·


DNA:  gcatccacacccgcaccacaacagaatcagcagtcaaataacaacaacactacctcggca
+1fr: ·A··S··T··P··A··P··Q··Q··N··Q··Q··S··N··N··N··N··T··T··S··A·


DNA:  aattctaaatctagccagcagaatcctccacagatatatccctggatgaagcgtgtccac
+1fr: ·N··S··K··S··S··Q··Q··N··P··P··Q··I··Y··P··W··M··K··R··V··H·


DNA:  ctcggacaaagtactgttaacgctaacggtgaaacgaaacgtcaacggacgtcctatacg
+1fr: ·L··G··Q··S··T··V··N··A··N··G··E··T··K··R··Q··R··T··S··Y··T·


DNA:  agataccaaactctggaactggaaaaagaatttcatttcaacaggtacctgacaaggcgg
+1fr: ·R··Y··Q··T··L··E··L··E··K··E··F··H··F··N··R··Y··L··T··R··R·


DNA:  aggaggatagaaatagcacacgctctctgcctaactgaaaggcaaatcaaaatctggttt
+1fr: ·R··R··I··E··I··A··H··A··L··C··L··T··E··R··Q··I··K··I··W··F·


DNA:  caaaacagaaggatgaaatggaagaaagaacataaaatggcatcgatgaacgtcattccg
+1fr: ·Q··N··R··R··M··K··W··K··K··E··H··K··M··A··S··M··N··V··I··P·


DNA:  taccactaccacatgtcacaaccttacggtaacccttaccagtttactcatctgaccacc
+1fr: ·Y··H··Y··H··M··S··Q··P··Y··G··N··P··Y··Q··F··T··H··L··T··T·


DNA:  taa
+1fr: ·*·

**Figure S1: *Sex Combs Reduced* coding sequence of *Rhagovelia antilleana.*** The yellow highlight represents the region targeted by the RNAi.

DNA:  atgaactcgtattttgagcagacgggtttctacggggctcatcatcaccagggcggctca
+1fr: ·M··N··S··Y··F··E··Q··T··G··F··Y··G··A··H··H··H··Q··G··G··S·


DNA:  gctcatcatcaccacgaacaagcggcggcctatagatttccactcagcctaggaatgtca
+1fr: ·A··H··H··H··H··E··Q··A··A··A··Y··R··F··P··L··S··L··G··M··S·


DNA:  ccatacgcctcaagtcagcaccatcatcacggactccaagctcccagaccccaagactct
+1fr: ·P··Y··A··S··S··Q··H··H··H··H··G··L··Q··A··P··R··P··Q··D··S·


DNA:  ccttacgacgcctctgtcgctgctgcctgcaaattatactcggcatcagcagactctgcc
+1fr: ·P··Y··D··A··S··V··A··A··A··C··K··L··Y··S··A··S··A··D··S··A·


DNA:  gcctccaactacggatcccccgtggccaaacccgactgtagcaagaccgagggacacacg
+1fr: ·A··S··N··Y··G··S··P··V··A··K··P··D··C··S··K··T··E··G··H··T·


DNA:  aacgggtacggtaaagaagtgtgggggcgtgagtcgggcggcaggtacgggggtctggcc
+1fr: ·N··G··Y··G··K··E··V··W··G··R··E··S··G··G··R··Y··G··G··L··A·


DNA:  ggttcgcccgagagcggtaggtcgtccgcacaacctctaggggcggccagtacccccggc
+1fr: ·G··S··P··E··S··G··R··S··S··A··Q··P··L··G··A··A··S··T··P··G·


DNA:  cccgcgtggaaccaatgcagcatcacacctacgtcaggtcagcctgccgtcgcaccctct
+1fr: ·P··A··W··N··Q··C··S··I··T··P··T··S··G··Q··P··A··V··A··P··S·


DNA:  cctttgcatcagcaagcgtctcagcacaccttctacccctggatggctatagcaggtaca
+1fr: ·P··L··H··Q··Q··A··S··Q··H··T··F··Y··P··W··M··A··I··A··G··T·


DNA:  ttcgttggagcgaatgggttaaggcggagaggtcggcagacgtataccaggtaccaaaca
+1fr: ·F··V··G··A··N··G··L··R··R··R··G··R··Q··T··Y··T··R··Y··Q··T·


DNA:  ctagaattggaaaaagagttccacaccaaccactacctgacaaggcggcggaggatagag
+1fr: ·L··E··L··E··K··E··F··H··T··N··H··Y··L··T··R··R··R··R··I··E·


DNA:  atggctcacgctctctgtcttacagaaagacagataaaaatatggttccaaaatcggagg
+1fr: ·M··A··H··A··L··C··L··T··E··R··Q··I··K··I··W··F··Q··N··R··R·


DNA:  atgaagctcaagaaagaaatacaggcgataaaggagctcaacgaacaggagaaacaagcg
+1fr: ·M··K··L··K··K··E··I··Q··A··I··K··E··L··N··E··Q··E··K··Q··A·


DNA:  caggcgcagaaagcggccgcggcagccgcagttattgccgctcaacagcaagaccattaa
+1fr: ·Q··A··Q··K··A··A··A··A··A··A··V··I··A··A··Q··Q··Q··D··H··*·

**Figure S2: *Ultrabithorax* coding sequence of *Rhagovelia antilleana.*** The yellow highlight represents the region targeted by the RNAi.


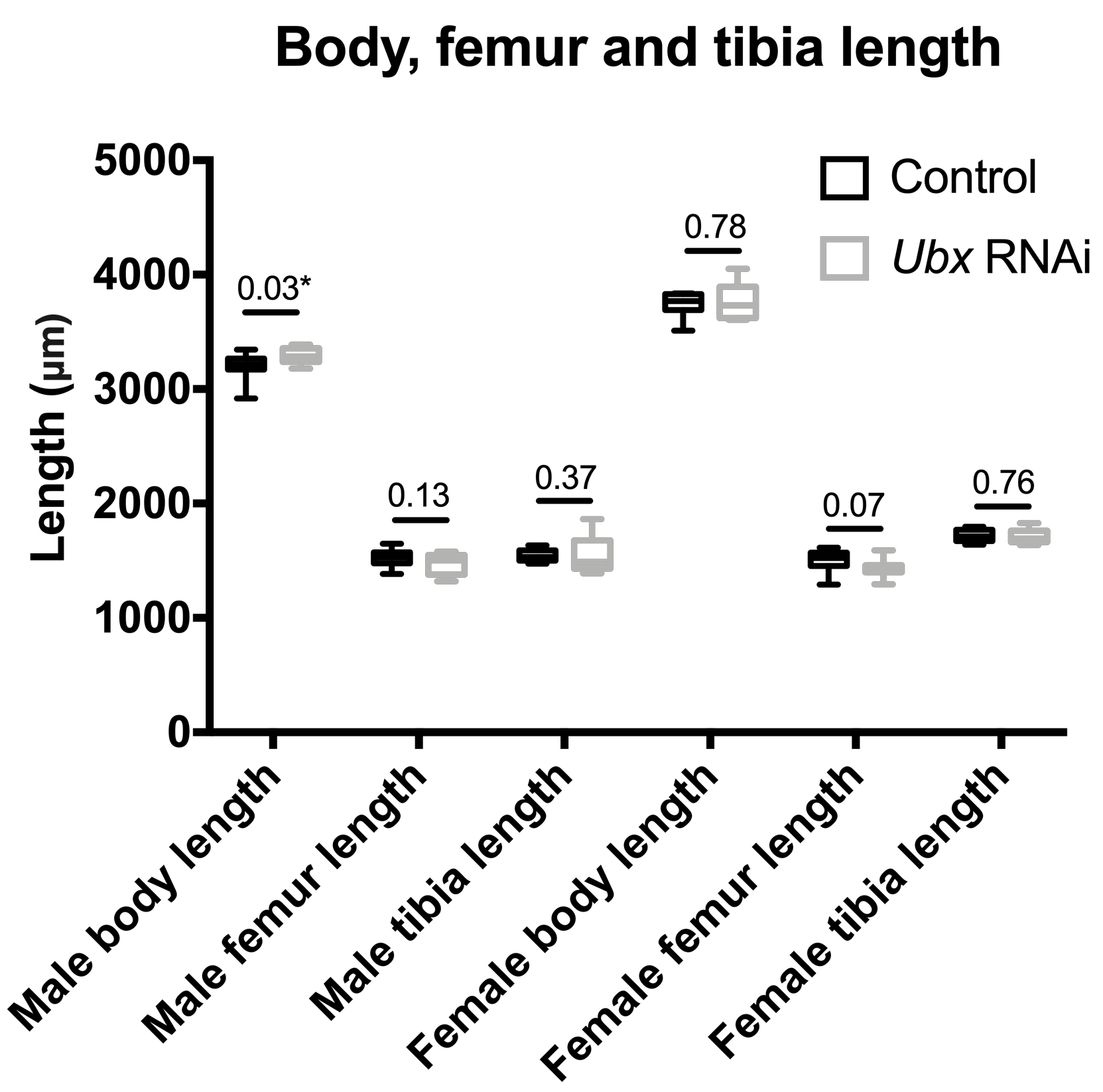


**Figure S3: Comparison of body, femur and tibia length between Ubx RNAi and control.** There is no clear differences in the length of these different body parts between *Ubx* RNAi and control individuals in both males and females. P-values are indicated on top of each comparisons. Effectives: *yfp* control males n=21, *Ubx* RNAi males n=9, *yfp* control females n=12, *Ubx* RNAi females n=10.

| Used for : | Gene | | Primer | Sequence 5’ to 3’ |
| --- | --- | --- | --- | --- |
| Cloning | *Sex Combs Reduced* | | Rantilleana-scr-fw7 | GAATACGCTCAGCTCGGTTC |
| Cloning | *Sex Combs Reduced* | | Rantilleana-scr-rev7 | TTGCCGAGGTAGTGTTGTTG |
| Cloning | *Ultrabithorax* | | SAB-Ubx-ATG-F | ATGAATTCTTACTTTGAGCAGACGGGT |
| Cloning | *Ultrabithorax* | | Ubx-HDR6 | CTGCTATAGCCATCCAGGGGTAGAA |
| dsRNA | *Sex Combs Reduced* | | Rantilleana-scr-fw-T7 | **TAATACGACTCACTATAGGGAGACCAC**GAATACGCTCAGCTCGGTTC |
| dsRNA | *Sex Combs Reduced* | Rantilleana-scr-rev-T7 | | **TAATACGACTCACTATAGGGAGACCAC**TTGCCGAGGTAGTGTTGTTG |
| dsRNA | *Ultrabithorax* | SAB-Ubx-ATG-F-T7 | | **TAATACGACTCACTATAGGGAGACCAC**ATGAATTCTTACTTTGAGCAGACGGGT |
| dsRNA | *Ultrabithorax* | Ubx-HDR6-T7 | | **TAATACGACTCACTATAGGGAGACCAC**CTGCTATAGCCATCCAGGGGTAGAA |

**Table S1: List of primers.** Primers used to clone *Scr* and *Ubx* genes and to product dsRNA (T7 promoter sequence in bold) in *R. antilleana*.

| **Target gene** | **Number of injected individuals** | **Number of adult male obtained** | **Number of adult female obtained** | **Wild type phenotype obtained** | **RNAi phenotype obtained** |
| --- | --- | --- | --- | --- | --- |
| ***yfp* (control)** | 297 | 28 | 43 | 71 | 0 |
| ***Scr*** | 47 | 8 | 13 | 0 | 21 |
| ***Ubx*** | 348 | 22 | 27 | 0 | 49 |

**Table S2: Counting of nymph injections and phenotypes obtained in adults for *yfp* control, *Scr* and *Ubx* RNAi.** Our RNAi experiment have a high level of penetrance for both *Scr* and *Ubx* with all adults obtained that show phenotypes compared to the control condition.
